# Centrally mediated responses to NMES are influenced by muscle group and stimulation parameters

**DOI:** 10.1038/s41598-024-75145-2

**Published:** 2024-10-22

**Authors:** Timothée Popesco, Quentin Gardet, Jonathan Bossard, Nicola A. Maffiuletti, Nicolas Place

**Affiliations:** 1 Institute of Sport Sciences, University of Lausanne, Lausanne, 1015 Switzerland; 2 Human Performance Lab, Schulthess Clinic, Zurich, Switzerland

**Keywords:** Extra force, Sustained EMG activity, NMES, Pulse duration, Stimulation frequency, Inter-muscle comparison, Neurophysiology, Neural circuits

## Abstract

Wide-pulse high-frequency neuromuscular electrical stimulation (WPHF NMES) can generate a progressive increase in tetanic force through reflexive recruitment of motor units, called extra force. This phenomenon has previously been observed on different muscle groups, but little is known on potential inter-muscle differences. We compared extra force and sustained electromyographic (EMG) activity induced by NMES between plantar flexors, knee extensors, elbow flexors and within muscle groups using pulse durations of 0.2, 1 and 2 ms and stimulation frequencies of 20, 50, 100 and 147 Hz. Extra force production and sustained EMG activity were higher for plantar flexors compared to elbow flexors at all tested parameters (except 0.2 ms for extra force). When compared to elbow flexors, extra force of the knee extensors was only higher at 100 Hz and with 1 ms while sustained EMG activity was higher at all frequencies with pulse durations of 0.2 and 2 ms. Peripheral nerve architecture as well as muscle typology and function could influence the occurrence and magnitude of centrally-mediated responses to NMES. The present findings suggest that the use of wide-pulse high-frequency NMES to promote reflexive recruitment seems to be more pertinent for lower limb muscles, plantar flexors in particular.

**Supplementary Information:**

The online version contains supplementary material available at 10.1038/s41598-024-75145-2.

## Introduction

Neuromuscular electrical stimulation (NMES) is an effective training/treatment strategy for enhancing, preserving and restoring neuromuscular function in a variety of patient populations^1^, such as during/after a period of disuse due to illness, injury or surgery. Motor unit (MU) recruitment in response to NMES is random, synchronous and spatially fixed/superficial as compared to voluntary muscle contractions^2^. The main consequences of such peculiar and quite unphysiological recruitment pattern are represented by higher fatigability^3,4^ and discomfort^5^ compared to voluntary contractions.

There is growing evidence that manipulation of conventional stimulation parameters, i.e., pulse durations of 0.1–0.5 ms and pulse frequencies of 50–100 Hz^6^, may partly overcome these limitations. For instance, centrally-mediated responses could be increased by using wide-pulse high-frequency (WPHF) NMES. This emerging NMES modality is characterized by pulses of long duration (1 ms) delivered at a relatively high frequency (80–100 Hz)^7,8^. Wide pulses lead to a preferential recruitment of Ia sensory axons because of their longer strength-duration time constant and lower rheobase than motor axons^9,10^, which may promote MU reflexive recruitment. Furthermore, high stimulation frequencies facilitate the temporal summation of post-synaptic excitatory potentials and reflexively recruit spinal motoneurons through Ia afferents^11^, which may allow a progressive force enhancement during the stimulation train (the so-called ‘extra force’). An advantage of WPHF NMES over conventional NMES is that relatively low stimulation intensities are required to limit the antidromic collision that in turn depresses the reflexive response. By stimulating at intensities expected to generate only ~ 10% of the maximal voluntary contraction (MVC) force, WPHF NMES may induce in some individuals (responders) an extra force that can reach up to 80% of the MVC force for the plantar flexors^12^. However, the proportion of responders to WPHF NMES for this muscle group is only ~ 50%^7,13,14^, and it may well be that stimulation parameters other than those typically used for WPHF NMES (1 ms and 100 Hz) may further enhance the force response. The centrally-mediated extra force is usually accompanied by a prolongation of the surface electromyographic (EMG) activity after cessation of the stimulation train, also called ‘sustained EMG activity’^13^. This phenomenon is supposed to reflect the strength of persistent inward current (PIC) activation^12,14^, which would facilitate MU recruitment through the central pathway.

Plantar flexors are implied in posture and composed of *soleus*, *gastrocnemius medialis* and gastrocnemius *lateralis* muscles. The *soleus* muscle—the main contributor to plantar flexion at a knee angle of 90°^15,16^—is predominantly composed of type I muscle fibers^17^ which contain more neuromuscular spindles than type II fibers^18^. This greater amount of neuromuscular spindles translates into larger spinal reflexes^19,20^. As the production of centrally-mediated forces also depends on the depolarization of Ia afferents, variation in pulse duration should impact more the muscles depending on their density in Ia afferents. Higher stimulation frequencies could also influence the response as some motoneurons start firing supposedly due to the action potentials generated in Ia afferents through temporal summation^11^ and also via an increase in corticospinal excitability that has been observed especially with a frequency of 100 Hz^21^. So far, WPHF NMES has been mostly delivered to the plantar flexors^8,12,22,23^ as they have presumably stronger centrally-mediated responses compared to other muscle groups^24^. Attempts have been performed to generate extra force using pulses of 1 ms at 100 Hz in other muscle groups such as knee extensors^25^ and elbow flexors^26^, but no comparison was made between different muscle groups. Bergquist and colleagues compared six individuals who participated in two of their studies and observed that a simple increase in stimulation frequency from 15 to 100 Hz generated a higher increase in force on plantar flexors (48%) than on knee extensors (9%)^26^.

The impact of stimulation frequency on evoked force and sustained EMG activity has mainly been investigated on plantar flexors^24,27^ and knee extensors^28,29^, but with a limited range of frequencies (20–100 Hz). The influence of pulse duration on evoked force and sustained EMG activity has been widely examined on plantar flexors, knee extensors^30–32^ and elbow flexors^33^, mostly for pulses ≤ 1 ms. Several other studies did not assess independently the effect of stimulation frequency vs. pulse duration as they only compared two extreme combinations: low frequency-narrow pulse vs. high frequency-wide pulse^7,34–39^. Thus, the influence of various stimulation frequencies and pulse durations on centrally-mediated responses has never been systematically assessed.

The primary aim of the present study was to compare extra force and sustained EMG activity in response to NMES between plantar flexors, knee extensors and elbow flexors with various pulse durations and frequencies. It was hypothesized that plantar flexors would show larger centrally-mediated responses to NMES, observed through higher extra force and sustained EMG activity, than knee extensors and elbow flexors, especially with pulse durations ≥ 1 ms. The secondary aim was to compare extra force and sustained EMG activity between different pulse durations and frequencies for each of these muscle groups. It was hypothesized that higher frequencies and longer pulses would evoke larger centrally-mediated responses, as reflected by higher extra forces and greater sustained EMG activities.

## Methods

### Subjects

Sixteen healthy participants (2 women, 14 men: 29 ± 6 years; 74 ± 11 kg; 177 ± 6 cm) volunteered to participate in this study. They were free from any neuromuscular disorder or any muscle injury on the tested muscles. The study protocol was approved by the Ethics Committee of the Vaud canton (protocol 2016 − 00563) and was in accordance with the latest update of the Helsinki Declaration. Informed consent was obtained from all participants included in the study.

### Experimental protocol

All participants were familiarized with the different procedures at least 24 h before the first experimental session. Familiarization included three to five MVCs and two 10-s WPHF NMES stimulation trains at random pulse durations (0.2, 1 and 2 ms) and frequencies (20, 50, 100 and 147 Hz) for each muscle group considered (plantar flexors, elbow flexors and knee extensors). The experiments were performed unilaterally on the right limb for all participants and under isometric conditions. The study included one experimental session per muscle group for a total of three sessions performed in a randomized order and separated by 1–7 days. NMES was delivered over the muscle belly instead of to the nerve trunk as (i) discomfort has been shown to be lower for muscle belly stimulation^12,40^,(ii) elbow flexion force cannot be evoked through brachial plexus stimulation^37^ and (iii) NMES is delivered to the muscle belly in clinical settings^1^.

**Fig. 1:**
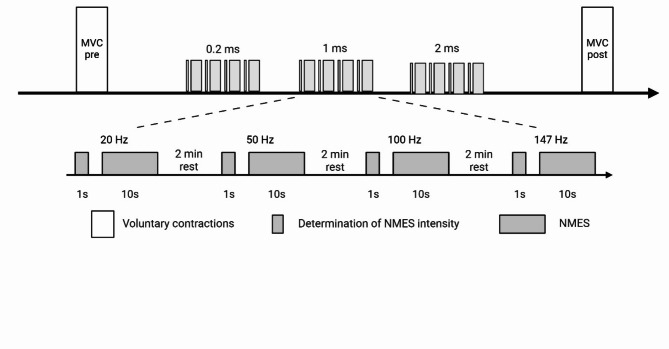
Experimental protocol. The different stimulation frequencies and pulse durations are displayed in the ascending order but were in fact randomized. MVC = maximal voluntary contraction; NMES = neuromuscular electrical stimulation.

The experimental protocol was similar for the three muscle groups (Fig. 1). After a warm-up consisting of submaximal voluntary contractions (20–80% of the estimated MVC force), participants performed several MVCs (with a minimum of 2 and a maximum of 5) until when the coefficient of variation of two trials was < 5%^12^. Rest periods of 60 s were observed between trials. Participants were asked to produce their maximal force within 1–2 s and to hold it for about 3 s. Then, one pulse duration amongst the three tested (0.2, 1 and 2 ms) was randomly chosen and when the pulse duration had been selected, one stimulation frequency out of the four tested (20, 50, 100 and 147 Hz) was also randomly picked to start. Trains of 1 s were first evoked to find the intensity (mA) of stimulation eliciting 10% of the MVC force. Once it was reached, a 10-s WPHF NMES train was delivered at this intensity. After 2 min of rest, the subsequent 1-s and 10-s trains were delivered at the next frequency. Right after the stimulation, participants were asked to rate their perceived discomfort. When all frequencies were tested for this pulse duration, the same process was followed for the next pulse durations. The order in which the various frequencies and pulse durations were tested was counterbalanced. Finally, two MVCs were performed (separated by 1 min of rest) to evaluate whether the entire protocol resulted in fatigability.

### Data collection and analysis

#### Electrical stimulation

**Fig. 2:**
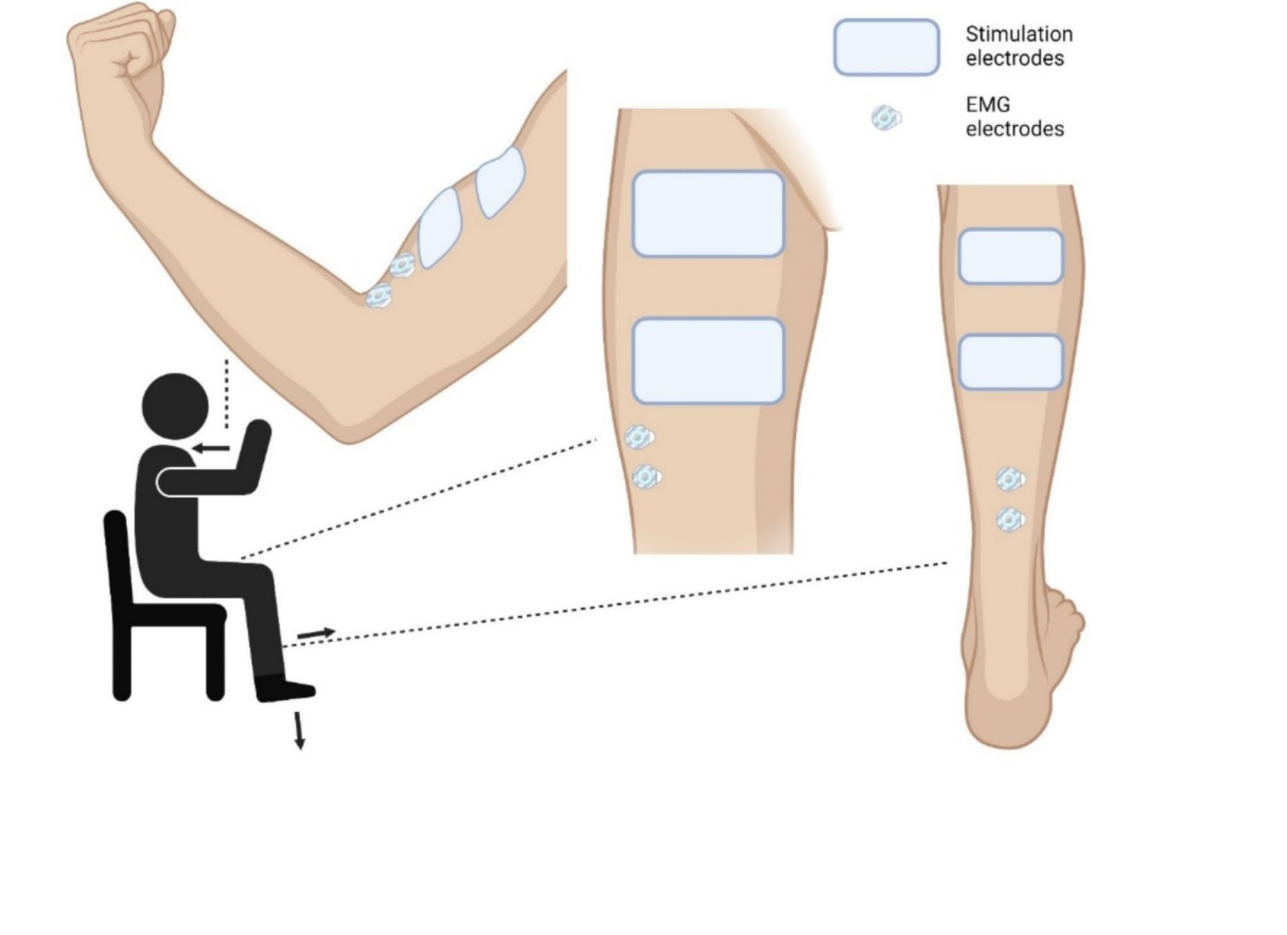
Position of the participant and placement of the stimulation and electromyographic (EMG) electrodes on each muscle group (elbow flexors, knee extensors, plantar flexors from left to right). Arrows represent the direction of force production.

NMES was delivered by a constant-current stimulator (Digitimer, DS7AH, Hertfordshire, UK) transcutaneously using two electrodes per muscle. Single stimuli (monophasic pulses of 1 ms at 400 V) were first delivered using a stimulation pen (Compex, Ecublens, Switzerland) to locate the motor points of the different muscles, i.e. the site where the strongest response was induced by the weakest current^41^. Electrodes were positioned following previous recommendations and individualized determination of motor points. First, the specific area was identified according to previous works^22,42,43^. For plantar flexors, the anode and cathode (5 cm x 10 cm, Uni-Patch R series, Covidien, Dublin, Ireland) were placed respectively on the widest part of both gastrocnemii muscles (~ 4 cm below the popliteal fossa) and on the *soleus* just below the *gastrocnemii*^22^. For knee extensors, electrodes (8 cm x 13 cm, Stimex, Regensdorf, Switzerland) were placed on the proximal part of the *quadriceps*, 5–10 cm below the inguinal crease and 5–10 cm above the superior border of the patella over the muscle belly of *vastus lateralis*,* rectus femoris*,* and vastus medialis*^42^. For elbow flexors, electrodes (5 cm x 5 cm, Myotrode Premium, Globus, Bressanone, Italia) were placed on the *biceps brachii* at ~ 35% of the distance from the elbow crease to the acromion and covering almost the entire muscle belly^43^, more precisely on the proximal part of the short portion and on the distal part of the long portion of the *biceps brachii* (Fig. 2). Then, single stimuli (monophasic pulses of 1 ms at 400 V) were delivered using a stimulation pen (Compex, Ecublens, Switzerland) to precisely localize the motor points of the different muscles, i.e. the site where the strongest response was induced by the weakest current^41^.

At the end of each 10-s stimulation train, a visual analog scale (VAS) was given to the participant to rate the discomfort induced by NMES. The scale ranges from 0 (not painful) to 10 (extremely painful).

#### Force

Force was assessed using muscle-specific custom-made dynamometers. Plantar flexor force was measured using a foot pedal connected to a strain gauge (capacity: 110 Nm, Vishay Micro Measure, Raleigh, USA). Participants were seated on a stool with the knee bent at 90° and the ankle in neutral position (0° of plantar flexion). The distal part of the thigh (just above the patella) was held by a horizontal bar mounted on the foot pedal to avoid vertical movements and contributions from other muscles. The foot was strapped to the pedal at the metatarsal and ankle levels. Knee extensor force was collected using a comfortable chair instrumented with a load cell (STS 2500 N, sensitivity 2.0005 mV/V and 0.0017 V/N; SWJ, Shenzhen, China). The load cell was securely attached just above the ankle with a custom mold and the knee angle was at 90°. The non-tested leg rested on a stool to avoid contact with the load cell. The participant was fixed to the chair with two shoulder straps crossing the trunk and another strap was fixed to the pelvis to limit extraneous upper body movements. Elbow flexor force was collected using an ergometer connected to a load cell (SAS 2000 N, sensitivity 1.998 mV/V and 0.0014 V/N; SWJ). Adjustments such as stool and handle height were set for the participant’s elbow to form a 90° angle and the handle height was optimally adjusted for grip and pull according to the participant feedback. Force signals were recorded at 1’000 Hz for knee extensors and 1’250 Hz for plantar flexors and elbow flexors using an analog-to-digital converter (MP150; BIOPAC, Goleta, CA, USA).

The highest (peak-to-peak) force developed during the best MVC attempt at the start of each session was considered as the MVC force. As already done in previous NMES studies^22–24,34,44^, extra force was calculated as the percent difference in evoked force between the 2nd second of the stimulation train (corresponding to initial force) and the last second of the train (final force), as follows: extra force = (final force – initial force)/initial force) x 100. Participants with an extra force superior to zero were classified as responders^22^. The mean absolute force between the 2nd second and the last second of the stimulation train was also calculated, considered as the mean evoked force and presented both in absolute and relative values. The highest mean force evoked for each participant was also calculated and referred to as best train mean force. All force data were recorded and analyzed with the Acqknowledge software (version 5, BIOPAC Systems, Goleta, CA, USA).

#### EMG activity

EMG activity was recorded with pairs of circular silver-chloride (Ag/AgCl) self-adhesive surface electrodes (1-cm diameter, Meditrace 100, Tyco, Markham, Canada), with an inter-electrode distance of 2 cm and placed according to SENIAM recommendations^45^ (Fig. 2). The skin was first shaved and cleaned with alcohol to minimize inter-electrode resistance. Electrodes were positioned lengthwise, parallel to muscle fibers, over the *soleus* muscle belly (Fig. 2) at a distance corresponding to 2/3 of the distance between the medial condyle of the femur and the medial malleolus for plantar flexors^22^, on the *vastus lateralis* muscle belly around 5 cm from the superior medial side of the patella along a line medially oriented at an angle of 50° with respect to the anterior superior iliac spine for knee extensors^46^, and on the *biceps brachii* muscle belly on the line between the medial acromion and the fossa cubit at 1/3 from the fossa cubit for elbow flexors. Reference electrodes were placed on the ipsilateral patella for plantar flexors and knee extensors and olecranon for elbow flexors. The EMG signals were amplified with a gain of 1000, collected at a sampling frequency of 5000 Hz for the *soleus*, 2000 Hz for the *vastus lateralis* and 2500 Hz for the *biceps brachii*, with a bandwidth of 10 to 500 Hz. EMG signals were recorded with the same analog-to-digitial converter used to record force (MP150; BIOPAC, Goleta, CA, USA).

The root mean square (RMS) value was calculated over a moving window of 250 samples to characterize both maximal EMG activity during MVC (EMGmax) and sustained EMG activity following NMES trains. The mean value of a 500-ms interval around peak force (250 ms before and 250 ms after) was used to quantify EMGmax. When the EMG traces immediately after a stimulation train showed the presence of sustained activity (as defined visually by the same experimenter), the RMS value reflecting sustained EMG activity was quantified over the time the sustained activity lasted (maximum 500 ms). If no sustained EMG was observed, the RMS value was quantified over 500 ms. This value was then normalized with respect to EMGmax for respective muscles. All EMG data were recorded and analyzed with the Acqknowledge software (version 5, BIOPAC Systems, Goleta, CA, USA).

### Statistics

Statistics were performed with Jamovi (version 2.2.5.0, Sydney, Australia) and data were plotted in graphical format using GraphPad Prism (GraphPad Software 8, Inc., San Diego, CA, USA). Q-q plots of residuals were computed for verification of their normality. For each stimulation frequency, a linear mixed model was used to compare extra force, sustained EMG activity, mean force, and VAS (dependent variables) with muscle group and pulse duration as factors. For each pulse duration, a linear mixed model was used to compare extra force, sustained EMG activity, mean force, and VAS (dependent variables) with muscle group and frequency as factors. These analyses were used to address the primary aim of the study. For the secondary aim, a linear mixed model was performed for each muscle group to compare extra force, sustained EMG activity, mean force, and VAS (dependent variables) with pulse duration and frequency as factors. Post hoc tests were then used when fixed effects were significant. MVC force data recorded before and after each session were compared using a Wilcoxon test. Correlations between extra force and sustained EMG activity were analyzed with Spearman correlation. Data are presented as mean ± SD. Statistical significance was set at an alpha level of *p* < 0.05. Details of statistical results (fixed effect significance, F values and degrees of freedom) can be found in Supplementary Tables 1 and 2.

## Results

**Fig. 3:**
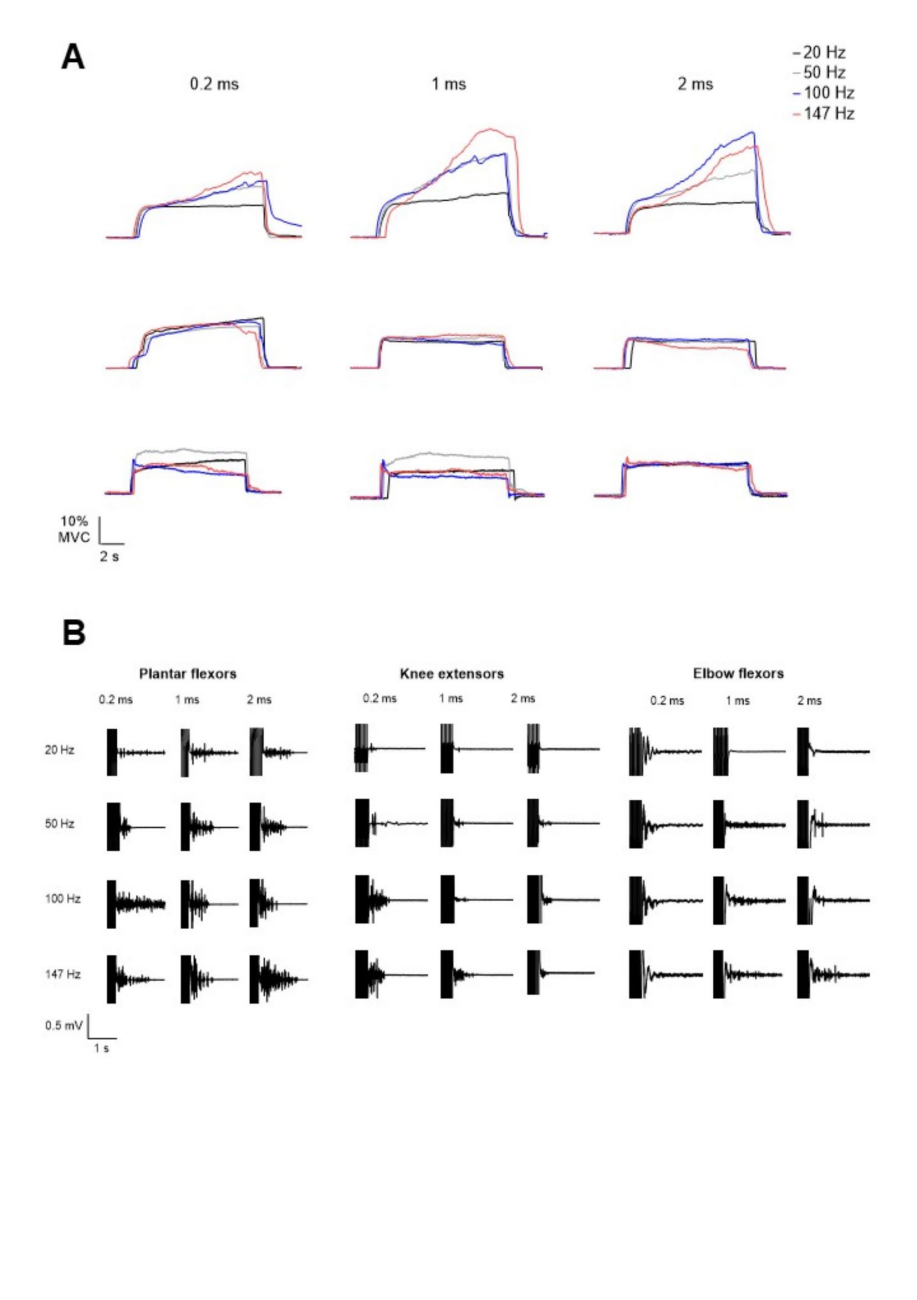
**A** Original traces of evoked force from a representative subject for plantar flexors (PF, upper panels), knee extensors (KE, middle panels) and elbow flexors (EF, lower panels). Force traces are presented for each pulse duration and frequency condition. **B** Original traces of sustained electromyographic (EMG) from a representative subject for the *soleus* (left), vastus lateralis (middle) and biceps brachii (right). EMG traces are presented for each pulse duration and frequency condition. Stimulation artifacts always precede sustained EMG activity, when visible.

Between all the participants, the MVC force measured before the stimulation trains reached 171 ± 44 N, 424 ± 133 N and 249 ± 52 N for plantar flexors, knee extensors and elbow flexors respectively. On average, the best train mean force was 23% (39 N), 22% (93 N) and 17% MVC force (42 N) and the mean evoked force averaged from all stimulation trains was 17% (30 N), 15% (65 N) and 12% MVC force (30 N) respectively for plantar flexors, knee extensors and elbow flexors (see original recordings displayed in Fig. 3). The participants who reached the highest mean forces when averaging all the stimulation trains were participant no. 3 for plantar flexors and knee extensors with an average mean force of 37% (65 N) and 45% MVC force (160 N) respectively, and participant no. 11 for elbow flexors with a mean force of 29% MVC force (72 N).

### Inter-muscle differences for stimulation frequency

**Fig. 4:**
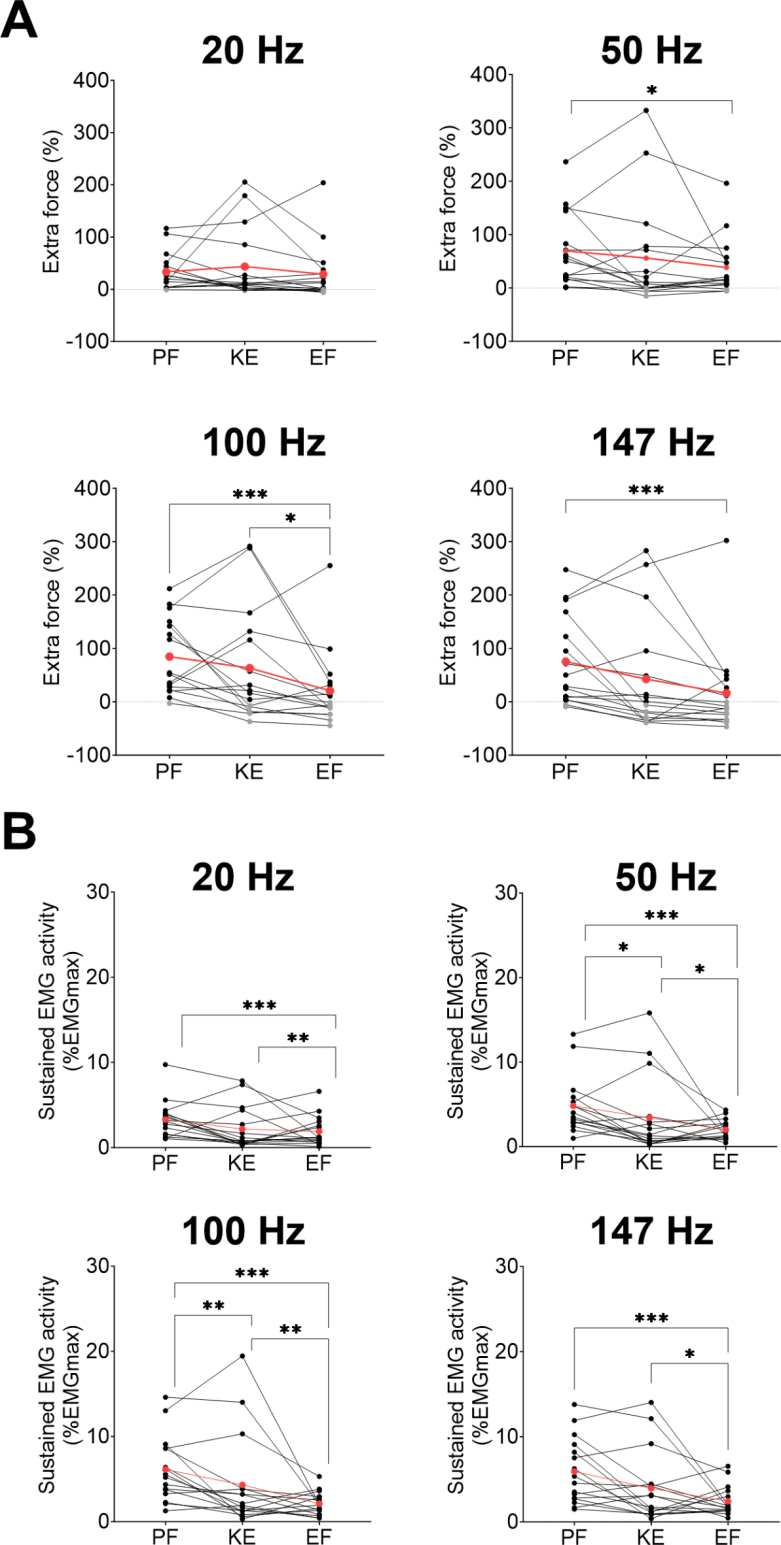
Comparison of extra force (**A**) and sustained electromyographic (EMG) activity (**B**) between muscle groups (PF: plantar flexors; KE: knee extensors; EF: elbow flexors) at each stimulation frequency. Individual and mean data (red) are displayed. For extra force, positive values representing responders are in black while negative values corresponding to non-responders are in grey. EMG activity was measured on *soleus* for plantar flexors, *vastus lateralis* for knee extensors and *biceps brachii* for elbow flexors. *p < 0.05, **p < 0.01, ***p < 0.001.

Extra force was significantly higher for plantar flexors than for elbow flexors at 50 Hz (69 ± 68 vs. 38 ± 53%, *p* = 0.025), 100 Hz (84 ± 71 vs. 21 ± 72%, *p* < 0.001) and 147 Hz (75 ± 84 vs. 16 ± 82%, *p* < 0.001), but not at 20 Hz (*p* = 0.649). For all frequencies, extra force was not significantly different between plantar flexors and knee extensors (*p* = 0.334 [20 Hz], *p* = 0.743 [50 Hz], *p* = 0.424 [100 Hz], *p* = 0.065 [147 Hz]). Extra force was significantly higher for knee extensors than elbow flexors at 100 Hz (63 ± 106 vs. 21 ± 72%, *p* = 0.012), but not at the other frequencies (*p* = 0.156 [20 Hz], *p* = 0.388 [50 Hz], *p* = 0.199 [147 Hz]). The proportion of responders at 20 Hz, 50 Hz, 100 Hz and 147 Hz was 94%, 100%, 100% and 88% for plantar flexors, 88%, 69%, 63% and 50% for knee extensors, and 69%, 81%, 44% and 38% for elbow flexors, respectively (Fig. 4A).

Sustained EMG activity was significantly higher for plantar flexors than for elbow flexors at 20 Hz (3.3 ± 2.2 vs. 1.9 ± 1.7% EMGmax; *p* < 0.001), 50 Hz (4.8 ± 3.3 vs. 2.0 ± 1.2% EMGmax; *p* < 0.001), 100 Hz (6.1 ± 3.8 vs. 2.1 ± 1.4% EMGmax; *p* < 0.001) and 147 Hz (5.9 ± 3.8 vs. 2.4 ± 1.7% EMGmax; *p* < 0.001) (Fig. 6). Sustained EMG activity was also significantly higher for plantar flexors compared to knee extensors at 50 Hz (4.8 ± 3.3 vs. 3.4 ± 4.6% EMGmax; *p* = 0.010), 100 Hz (6.1 ± 3.8 vs. 4.3 ± 5.5% EMGmax; *p* = 0.009) and 147 Hz (5.9 ± 3.8 vs. 3.9 ± 4.1% EMGmax; *p* = 0.003) but not at 20 Hz (*p* = 0.483). Finally, sustained EMG activity was significantly higher for knee extensors than for elbow flexors at 20 Hz (2.2 ± 2.5 vs. 1.9 ± 1.7% EMGmax; *p* = 0.005), 50 Hz (3.4 ± 4.6 vs. 2.0 ± 1.2% EMGmax; *p* = 0.011), 100 Hz (4.3 ± 5.5 vs. 2.1 ± 1.4% EMGmax; *p* = 0.001) and 147 Hz (3.9 ± 4.1 vs. 2.4 ± 1.7% EMGmax; *p* = 0.022, Fig. 4B).

The mean evoked force was significantly higher for plantar flexors and knee extensors compared to elbow flexors for frequencies ≥ 50 Hz (*p* ≤ 0.005). Mean force was significantly higher for plantar flexors compared to knee extensors at 100 and 147 Hz (*p* < 0.001). Discomfort was significantly higher for both knee extensors (2.1 ± 2.0) and elbow flexors (2.1 ± 1.5) than for plantar flexors (0.9 ± 0.9) at 20, 100 and 147 Hz (*p* < 0.001).

### Inter-muscle differences for pulse duration

**Fig. 5:**
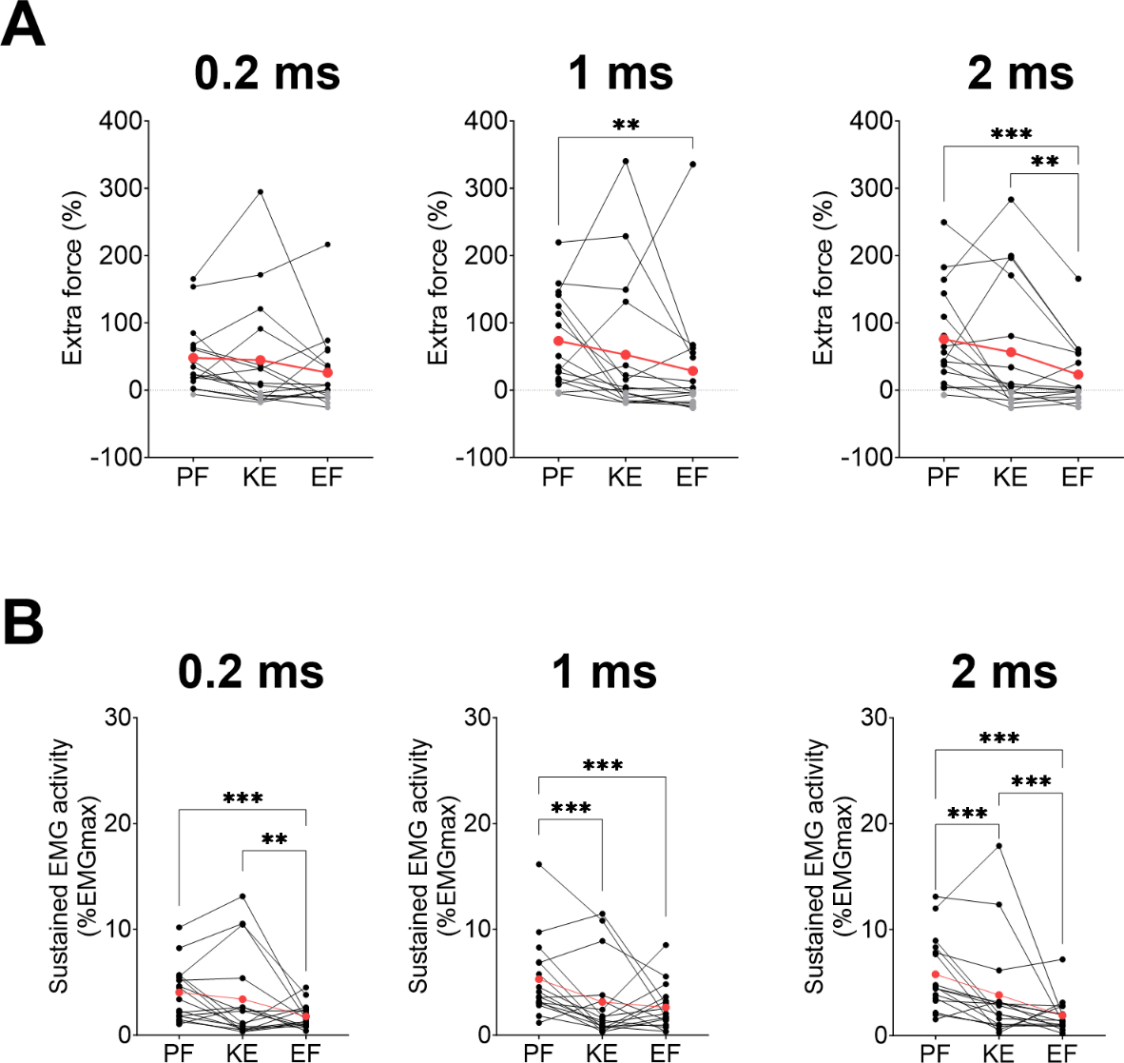
Comparison of extra force (**A**) and sustained electromyographic (EMG) activity (**B**) between muscle groups (PF: plantar flexors; KE: knee extensors; EF: elbow flexors) at each pulse duration. Individual and mean data (red) are displayed. For extra force, positive values representing responders are in black while negative values corresponding to non-responders are in grey. EMG activity was measured on *soleus* for PF, *vastus lateralis* for KE and *biceps brachii* for EF. **p < 0.01 and ***p < 0.001.

Extra force was significantly higher for plantar flexors than for elbow flexors with pulse durations of 1 ms (76 ± 74 vs. 23 ± 48%, *p* < 0.001) and 2 ms (73 ± 70 vs. 29 ± 88%, *p* < 0.001), but not with 0.2 ms (*p* = 0.064). Extra force was not significantly different between plantar flexors and knee extensors, but knee extensors showed a higher extra force than elbow flexors with 1 ms (56 ± 99 vs. 23 ± 48%, *p* = 0.002). The proportion of responders with pulse durations of 0.2, 1 and 2 ms was 94%, 94% and 88% for plantar flexors, 56%, 56% and 56% for knee extensors, and 63%, 63% and 50% for elbow flexors, respectively (Fig. 5A).

Sustained EMG activity was significantly higher for plantar flexors compared to elbow flexors with pulse durations of 0.2 ms (4.1 ± 2.6 vs. 1.8 ± 1.1% EMGmax; *p* < 0.001), 1 ms (5.3 ± 3.7 vs. 2.6 ± 2.1% EMGmax; *p* < 0.001), and 2 ms (5.8 ± 3.5 vs. 1.9 ± 1.6% EMGmax; *p* < 0.001). Sustained EMG activity of plantar flexors was also significantly higher than knee extensors with 1 ms (5.3 ± 3.7 vs. 3.1 ± 3.7% EMGmax; *p* < 0.001) and 2 ms (5.8 ± 3.5 vs. 3.8 ± 4.8% EMGmax, *p* < 0.001) but not with 0.2 ms. Knee extensors showed a higher sustained EMG activity than elbow flexors with 0.2 ms (3.4 ± 4.2 vs. 1.8 ± 1.1% EMGmax; *p* < 0.001) and 2 ms (3.8 ± 4.8 vs. 1.9 ± 1.6% EMGmax, *p* < 0.001), but not with 1 ms (Fig. 5B).

The mean evoked force was significantly higher for plantar flexors than both knee extensors and elbow flexors with 1 and 2 ms (*p* < 0.001), and for knee extensors compared to elbow flexors with 1 ms (*p* < 0.001). For all pulse durations, discomfort was significantly higher for both knee extensors (2.1 ± 2.0) and elbow flexors (2.1 ± 1.5) compared to plantar flexors (0.9 ± 0.8, *p* < 0.001).

**Fig. 6:**
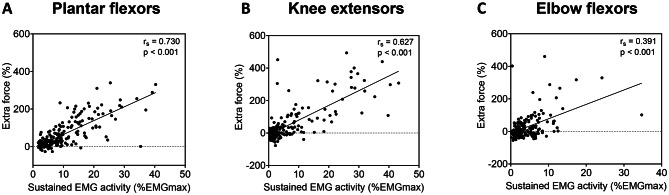
Correlation between extra force and sustained electromyographic (EMG) activity for *soleus* (**A**), *vastus lateralis* (**B**) and *biceps brachii* (**C**).

For each of the muscle groups, extra force and sustained EMG activity were significantly correlated (*p* < 0.001) (Fig. 6).

MVC force was slightly but significantly reduced at the end of the experimental session with a similar decline of 3.1%, 4.5% and 4.1% respectively for plantar flexors (*p* = 0.018), knee extensors (*p* = 0.047) and elbow flexors (*p* = 0.013).

### Inter-frequency differences by muscle group

For plantar flexors, extra force and sustained EMG activity were significantly higher at 50, 100 and 147 Hz compared to 20 Hz (*p* < 0.001). For knee extensors, sustained EMG activity was higher at 100 Hz and 147 Hz compared to 20 Hz while there was no difference between frequencies for elbow flexors (Table 1).

**Table 1:** Effect of stimulation frequency on extra force and sustained EMG activity by muscle group.

	20 Hz	50 Hz	100 Hz	147 Hz	*P* value
Extra force PF (%)	33.4 ± 39.0^bcd^	69.4 ± 75.2^a^	84.5 ± 78.4^a^	65.7 ± 92.3^a^	< 0.001
Extra force KE (%)	43.5 ± 90.2	56.0 ± 105.1	63.1 ± 118.7	42.4 ± 121.1	0.448
Extra force EF (%)	28.7 ± 56.1	38.3 ± 62.9	20.7 ± 82.6	16.2 ± 91.1	0.070
Sustained *soleus* EMG activity (% EMGmax)	5.0 ± 4.6^bcd^	7.2 ± 6.2^a^	9.3 ± 7.9^a^	9.2 ± 8.6^a^	< 0.001
Sustained *vastus lateralis* EMG activity (% EMGmax)	3.3 ± 4.7^cd^	4.5 ± 6.7	6.4 ± 8.5^a^	6.2 ± 7.0^a^	< 0.001
Sustained *biceps brachii* EMG activity (% EMGmax)	2.6 ± 2.4	3.2 ± 2.7	3.2 ± 2.6	3.4 ± 3.0	0.224

PF: plantar flexors; KE: knee extensors; EF: elbow flexors. a = different from 20 Hz, b = different from 50 Hz, c = different from 100 Hz, d = different from 147 Hz. EMGmax = maximal electromyographic activity.

### Inter-pulse duration differences by muscle group

For plantar flexors, extra force and sustained EMG activity were significantly higher with pulse durations of 1 and 2 ms compared to 0.2 ms. For knee extensors, extra force and sustained EMG activity were not different amongst pulse durations. For the elbow flexors, pulse duration had no influence on extra force, while sustained EMG activity was higher with 1 ms compared to both 0.2 ms and 2 ms (Table 2).

**Table 2:** Effect of pulse duration on extra force and sustained EMG activity by muscle group.

	0.2 ms	1 ms	2 ms	*P* value
Extra force PF (%)	48.0 ± 59.8^bc^	75.7 ± 83.2^a^	73.2 ± 80.4^a^	0.001
Extra force KE (%)	44.7 ± 100.9	56.5 ± 112.8	52.6 ± 114.1	0.620
Extra force EF (%)	26.0 ± 68.4	23.2 ± 57.7	28.6 ± 93.6	0.662
Sustained *soleus* EMG activity (% EMGmax)	6.2 ± 5.1^bc^	8.2 ± 7.9^a^	8.7 ± 8.1^a^	0.001
Sustained *vastus lateralis* EMG activity (% EMGmax)	5.0 ± 6.8	4.6 ± 6.4	5.7 ± 7.6	0.300
Sustained *biceps brachii* EMG activity (% EMGmax)	2.7 ± 2.1^b^	3.8 ± 3.3^ac^	2.7 ± 2.4^b^	0.002

PF: plantar flexors; KE: knee extensors; EF: elbow flexors. a = different from 0.2 ms, b = different from 1 ms, c = different from 2 ms. EMGmax = maximal electromyographic activity.

## Discussion

The primary aim of this study was to compare the centrally-mediated responses evoked by NMES between three different muscle groups with a range of pulse durations and stimulation frequencies. The amount of extra force was higher for plantar flexors than elbow flexors except for the lowest frequency (20 Hz) and the narrowest pulse duration (0.2 ms). This finding, which confirms our initial hypothesis, is further supported by the higher sustained EMG activity in plantar flexors compared with elbow flexors. Extra force was also higher for knee extensors than elbow flexors but only at the frequency of 100 Hz and pulse duration of 1 ms. No difference in extra force was observed between plantar flexors and knee extensors but sustained EMG activity was higher for plantar flexors in several conditions (50 Hz, 100 Hz, 1 ms and 2 ms). Our results also confirm that the application of high stimulation frequencies (100 and 147 Hz) and wide pulses (1 and 2 ms) to the lower limbs induced the highest extra forces.

### Centrally-mediated responses and muscle groups

For the plantar flexors, sensory feedback contributes to generate extra forces that can reach up to 80% MVC force during NMES despite initial force levels of only 5–10% MVC force^12^. In the present study, the “top” responder for each muscle group achieved a mean force of 37%, 45% and 29% MVC force for the plantar flexors, knee extensors and elbow flexors, respectively, thereby, showing that evoked responses can be strongly modulated by the reflexive recruitment of motor units for the three tested muscle groups. The mean evoked force we observed for plantar flexors (17% MVC force) and elbow flexors (12% MVC force) were comparable to the results of Neyroud^12^ and Blouin^26^ who reported mean evoked force values of respectively ~ 16% and ~ 12% MVC force with an equivalent initial target of 10% MVC force. However, our results showed higher extra force for the knee extensors as compared to the results of Bergquist (2012) (15 vs. 10% MVC force). When comparing muscle groups, three potential factors may explain the heterogeneous responses to NMES: peripheral nerve architecture, muscle typology and muscle function.

Central mechanisms are thought to be responsible for the presence and magnitude of extra force via the recruitment of spinal motoneurons by the NMES-evoked sensory volley^23,24^. Then, the inter-muscle and inter-individual variations of extra force find their origin in peripheral differences as the amount of sensory fibers is key for extra force production^34^ and their proportion can widely vary between muscles and individuals^11,17^. Knowing that axon depolarization depends on its size as well as its distance from the electrode, interindividual differences in the depth of intramuscular nerve branches as well as the architecture of the peripheral nerve and its terminal branches^47,48^ might affect extra force production. The inter-muscle differences could be influenced by this neuronal architecture beneath the stimulation electrodes. In fact, our results could be explained by a deeper localization of nerve endings in *quadriceps* and *biceps brachii* muscles as compared to *triceps surae*. It has been observed that densest intramuscular nerve arborization is close to the surface of the skin in *gastrocnemii* and *soleus* muscles^49^, which might explain the higher levels of sustained EMG activity for plantar flexors in response to NMES. Moreover, subcutaneous layer thickness might impact NMES effectiveness as the closer location of nerve endings is thought to facilitate the reflexive recruitment of MUs. It has been observed that this subcutaneous layer is greater on knee extensors (*vastus lateralis*: 8 mm, *vastus medialis*: 7 mm, *rectus femoris*: 10 mm) compared to plantar flexors (*gastrocnemii*: 6.5 mm)^50^ which might explain the higher sustained EMG activity for the latter with 1, 2 ms, 50 and 100 Hz. In addition, muscle shortening during isometric contraction could reduce the depolarization of afferent fibers due to the change in the distance between the nerve endings and the electrodes. As shortening of pennate muscles (*soleus*,* gastrocnemii*, *vastus medialis*, *vastus* *lateralis* and *rectus femoris)* is lower than fusiform muscles (*biceps brachii*)^51^, this may contribute to the lowest extra force observed for elbow flexors in our study.

The neuromuscular spindle density hypothesis suggests that a greater amount of Ia afferents would allow for a greater centrally evoked force production^11^. It has also been shown that neuromuscular spindles – innervated by Ia afferents – are located in type I muscle fibers^18^. Thus, a greater percentage of type I muscle fibers would result in higher extra force levels. Our results support this hypothesis as the anti-gravity plantar flexors show a higher percentage of type I muscle fibers as compared to elbow flexors (*medial gastrocnemius*: 51% type I; *soleus*: 86% type I vs. *biceps brachii*: 58% type II muscle fibers)^17^. The similarity in muscle typology between knee extensors (*rectus femoris*: 71% type II, *vastus medialis* 67% type II, *vastus lateralis* 56% type II muscle fibers) and elbow flexors can also explain the comparable level of extra force between the two muscle groups^17^. The lower extra force production of elbow flexors might be explained by the higher proportion of type II muscle fibers in this muscle group as compared with plantar flexors and knee extensors^17^. Moreover, the deep localization of type I fibers in the knee extensors – potentially not or poorly activated by low-intensity NMES – can explain the lower sustained EMG activity observed for this muscle group^17,52,53^. When comparing results obtained on knee extensors^25^ and elbow flexors^26^ to plantar flexors using the same stimulation parameters^12,34,37,39,54,55^, similar conclusions can be drawn which support the hypothesis of a higher central contribution for muscles with a slower typology. The higher sustained EMG activity for plantar flexors and knee extensors compared to elbow flexors using 0.2 and 2 ms and at all frequencies (Figs. 4 and 5) could be explained by the contribution of PICs in the generation of centrally-mediated force in response to NMES. Indeed, PICs are mainly responsible for posture^56^ and are believed to be more present in smaller motoneurons even though recent studies comparing their estimates on different muscles are not unanimous on this point^57–61^.

Muscle function can play a role in the response to WPHF NMES; neuromuscular spindle density abundance for instance was positively correlated with functional indices during walking^62^. PIC activation is also expected be greater in plantar flexors and knee extensors compared to elbow flexors considering that the lower limb muscles are both involved in posture^63^ and that PICs play an important role in the maintenance of posture^64,65^. The characteristics of sustained EMG activity supports the hypothesis of a strong influence of PICs on centrally-mediated forces as they have been shown to amplify and prolong motoneuron discharge^66^. A positive correlation was indeed observed in this study between extra force and sustained EMG activity for all three muscle groups (Fig. 6) which extends previous findings obtained on plantar flexors only^22^ and confirms the potential link between the amplification of force production and the prolongation of motoneuron discharges even on other—less ‘responsive’—muscle groups.

Although discomfort was generally greater for knee extensors and elbow flexors compared to plantar flexors, VAS scores were in accordance with previous studies in which the same target force was used^12,37^. Contrary to what was reported by Gravholt and colleagues^28^, we observed no negative correlation between VAS and extra force or sustained EMG activity, i.e. there was no detrimental effect of discomfort on the evoked response. Although the nociceptive input induced by the stimulation might decrease the amplitude of reflex responses as it is the case for H reflex^67^, our results suggest that MU reflexive recruitment was apparently not influenced by the discomfort under the present experimental conditions, i.e. with a low initial target force. Finally, the extent of muscle fatigability, assessed with the reduction in MVC force, was significant but relatively low (– 4%) and comparable for the three different muscle groups. It is therefore unlikely that the distinct behavior we observed for different muscle groups was influenced by the development of muscle fatigability.

### Centrally-mediated responses and stimulation parameters

Despite large inter-individual differences in centrally-mediated responses at certain stimulation frequencies, the results showed overall significantly higher extra forces for high frequencies when NMES was applied to the plantar flexors. This can be explained by enhanced temporal summation of post-synaptic excitatory potentials, which reflexively activate spinal motoneurons through Ia afferents^11^. This temporal summation of postsynaptic excitatory potentials as well as self-sustained motoneuron firing induced by PICs are thought to be responsible for the sustained EMG activity after the stimulation train^7,11,13,23,44^, which can be considered as an indirect indicator of prolonged motoneuron discharge^12,22^. These results confirm those reported by Bergquist et al.^54^, who showed similar extra forces was at 100 Hz and 200 Hz but no extra force at 25 Hz. It is thought that axonal hyperpolarization may reduce the ability to recruit sensory axons and thereby lowers the sensory volleys to the central nervous system^68^, which may explain why force does not further increase for frequencies above 100 Hz. From our results, the frequency of 100 Hz seems to be the optimal for plantar flexors to maximize the additional force through the central recruitment of MUs. The increase in corticospinal activity mediated by peroneal nerve stimulation has been found to be greater with a frequency of 100 Hz as compared to 20, 50 and 200 Hz^21^. Therefore, an increase of the excitability within the corticospinal pathway in response to plantar flexor stimulation could also explain the greater extra force at this frequency. Extra force was also observed in response to low stimulation frequencies such as 20 Hz, as previously reported in former studies^8,69^, thereby confirming that reflexive recruitment of spinal motoneurons can also occur at low stimulation frequency. Extra force would thus not be generated above a frequency threshold but would rather follow a dose-response effect. However, this potential dose-response effect was not observed on knee extensors and elbow flexors as the extent of extra force did not differ between any frequency condition (Table 1). It indicates that, even though differences were observed between frequencies on plantar flexors, the use of higher frequencies is not of primary importance to maximize extra force for these muscle groups.

Concerning pulse duration, plantar flexor extra force was larger with pulses of 1–2 ms compared to 0.2 ms, as already observed in several studies^23,24,34,37,54^. Indeed, narrow pulses (0.05–0.4 ms) would preferentially activate motor axons, while wider pulses (≥ 0.5 ms) would recruit relatively more sensory axons due to a longer strength-duration time constant and lower rheobase than motor axons^10,70^. In the present study, the number of responders within muscle groups and across pulse durations was stable, but, for plantar flexors, extra force generation was more variable when using narrower compared to wider pulses, indicating that pulse duration rather affects the magnitude of the response in responders than the responder status (i.e. the presence of extra force for a given individual). The greater sustained EMG activity we observed for wide compared to narrow pulses confirms the findings of a previous study in which two pulse durations (0.2 and 1 ms) were compared for plantar flexors and elbow flexors^71^. In our study, 2-ms pulses did not generate higher extra force than 1-ms pulses, which suggests that pulses wider than 1 ms do not further increase the activation of sensory axons. In fact, while wider pulses (0.5 and 1 ms) have been shown to preferentially depolarize sensory axons compared to shorter pulses (0.05 ms)^54^, our results extend these findings by showing that pulses longer than 1 ms do not actually maximize extra force. For the knee extensors, modulating pulse durations did not influence the extent of extra force, which is consistent with previous findings^72^. This suggests that for this muscle group sensory fiber activation is less sensitive to pulse duration compared to the plantar flexors. This may again be explained by the deep localization of type I muscle fibers in the quadriceps^17,52,53^. In fact, low-intensity (superficial) stimulation might not be enough to reach Ia afferents inside neuromuscular spindles located deeply in the quadriceps, thereby leading to less central recruitment for this muscle group observed through differences in sustained EMG activity. No effect of stimulation frequency nor pulse duration was found on evoked force for elbow flexors, which contrasts with the results of Clair-Auger^33^. Their results were nevertheless obtained on hemiparetic patients with motoneuronal hyperexcitability which could have led to increased PIC strength and therefore a higher sensitivity to WPHF NMES.

Overall, our results suggest that the occurrence of extra force is dependent on pulse duration and stimulation frequency and varies widely due to peripheral nerve architecture and muscle typology and function.

### Limitations and perspectives

One of the limitations of this work is the absence of neurophysiological assessments that may have helped to explain the differences in centrally-mediated responses between conditions. However, previous studies did not find any correlation between the occurrence of extra force and H-reflex amplitude for the plantar flexors^55^, even though H-reflex depression has been observed after extra force production^34^. The techniques allowing to estimate PIC strength with high-density EMG^73^ could help to identify their contribution to the generation of extra force but this is not yet possible during evoked contractions. Another limitation of this study is the use of a unique initial target force (10% MVC force). This level was chosen as it allows to avoid the antidromic collision that can limit centrally-mediated force production, but the extra force between the different muscle groups might be preferentially produced at different initial force levels. However, even with this initial force level of 10% MVC, the magnitude of evoked forces was substantial for responders, attaining a mean force of 45% MVC force for the knee extensors of the best responder when averaging all the stimulation trains. If used repeatedly, these stimulation trains would potentially lead to strength gains thereby increasing NMES effectiveness in clinical and rehabilitation settings. To this purpose, the combination of voluntary contractions and NMES could also be beneficial to evoke higher extra forces as it has been observed to increase spinal excitability^74^ and thus it might optimize the extra force production^75^. The results of the present study suggest that the use of wide-pulse high-frequency NMES to generate centrally-mediated responses is more pertinent for lower limb muscles (especially plantar flexors) than for elbow flexors. These results also highlight the fact that frequencies higher than 100 Hz and pulses wider than 1 ms do not further facilitate centrally-mediated responses. Additional efforts are required to increase the centrally-mediated responses in NMES with the goal to reach higher training intensities and therefore improve the clinical applicability of WPHF NMES.

## Electronic supplementary material

Below is the link to the electronic supplementary material.

Supplementary Material 1

## Data Availability

The data that support the findings of this study are available from the corresponding author upon reasonable request.
