# Supplementary material for: Centrally mediated responses to NMES are influenced by muscle group and stimulation parameters
Source: Sci Rep. 2024 Oct 22;14:24918. doi: 10.1038/s41598-024-75145-2 (PMC11496505; doi:10.1038/s41598-024-75145-2)
Supplement: Supplementary file 1 — Supplementary Material 1 [file 41598_2024_75145_MOESM1_ESM.pdf]

Centrally-mediated responses to NMES are influenced by muscle group and stimulation parameters

Timothée Popesco[1], Quentin Gardet[1], Jonathan Bossard[1], Nicola A. Maffiuletti[2] and Nicolas Place[1]

[1]Institute of Sport Sciences, University of Lausanne, 1015 Lausanne, Switzerland

[2] Human Performance Lab, Schulthess Clinic, Zurich, Switzerland

Supplementary Information

Supplementary Table 1. Comparison of mean force and discomfort between muscle groups by stimulation frequency.

	PF	KE	EF	P value
Mean force 20 Hz (% MVC)	13.2 ± 3.8	11.9 ± 4.2	12.7 ± 5.4	0.071
Mean force 50 Hz (% MVC)	16.4 ± 7.3[c]	15.7 ± 10.7[c]	12.9 ± 4.4[ab]	0.005
Mean force 100 Hz (% MVC)	19.1 ± 11.3[bc]	15.8 ± 12.7[ac]	12.6 ± 8.3[ab]	<0.001
Mean force 147 Hz (% MVC)	18.1 ± 8.9[bc]	15.2 ± 10.8[ac]	12.1 ± 6.1[ab]	<0.001
Discomfort 20 Hz (VAS)	0.7 ± 0.8[bc]	1.8 ± 1.2[ac]	2.9 ± 2.0[ab]	<0.001
Discomfort 50 Hz (VAS)	0.8 ± 0.7	2.0 ± 1.8	2.9 ± 7.9	0.077
Discomfort 100 Hz (VAS)	1.0 ± 1.0[bc]	2.3 ± 2.8[a]	1.8 ± 1.1[a]	<0.001
Discomfort 147 Hz (VAS)	1.0 ± 0.9[bc]	2.1 ± 2.0[ac]	1.7 ± 4.2[ab]	<0.001

PF: plantar flexors; KE: knee extensors; EF: elbow flexors. a = different from PF, b = different from KE, c = different from EF.

Supplementary Table 2. Comparison of mean force and discomfort between muscle groups by pulse duration

	PF	KE	EF	P value
Mean force 0.2 ms (% MVC)	14.4 ± 8.2	14.1 ± 8.0	12.5 ± 5.6	0.115
Mean force 1 ms (% MVC)	17.6 ± 8.4^{bc}	14.8 ± 11.3^{ac}	12.6 ± 5.7^{ab}	<0.001
Mean force 2 ms (% MVC)	18.1 ± 9.8^{bc}	15.1 ± 12.6^{a}	12.6 ± 6.9^{a}	<0.001
Discomfort 0.2 ms (VAS)	0.6 ± 0.6^{bc}	1.9 ± 2.4^{a}	2.8 ± 6.9^{a}	0.011
Discomfort 1 ms (VAS)	0.9 ± 0.9^{bc}	2.1 ± 1.6^{a}	2.1 ± 1.5^{a}	<0.001
Discomfort 2 ms (VAS)	1.0 ± 0.9^{bc}	2.2 ± 1.8^{a}	2.0 ± 1.5^{a}	<0.001

PF: plantar flexors; KE: knee extensors; EF: elbow flexors. a = different from PF, b = different from KE, c = different from EF.

Supplementary Table 3. Linear mixed model statistics inter-frequency

Fixed effect tests		F	Degrees of freedom	P value
**Extra force 20 Hz**	*Muscle group*	0.61	2	0.547
	Pulse duration	1.06	2	0.349
	Muscle group x Pulse duration	0.25	4	0.907
**Extra force 50 Hz**	*Muscle group*	1.50	2	0.228
	Pulse duration	3.64	2	**0.029**
	Muscle group x Pulse duration	1.39	4	0.242
**Extra force 100 Hz**	*Muscle group*	0.17	2	0.840
	Pulse duration	10.14	2	**< 0.001**
	Muscle group x Pulse duration	0.25	4	0.908
**Extra force 147 Hz**	*Muscle group*	0.65	2	0.521
	Pulse duration	8.77	2	**< 0.001**
	Muscle group x Pulse duration	0.507	4	0.731
**Sustained EMG activity 20 Hz**	*Muscle group*	1.89	2	0.156
	Pulse duration	7.44	2	**< 0.001**
	Muscle group x Pulse duration	1.58	4	0.184
**Sustained EMG activity 50 Hz**	*Muscle group*	1.13	2	0.325
	Pulse duration	13.25	2	**< 0.001**
	Muscle group x Pulse duration	0.33	4	0.860
**Sustained EMG activity 100 Hz**	*Muscle group*	0.74	2	0.477
	Pulse duration	17.63	2	**< 0.001**
	Muscle group x Pulse duration	1.06	4	0.377
**Sustained EMG activity 147 Hz**	*Muscle group*	1.62	2	0.201
	Pulse duration	14.77	2	**< 0.001**
	Muscle group x Pulse duration	1.27	4	0.287

**Mean force 20 Hz**	*Muscle group*	2.67	2	0.073
	Pulse duration	0.56	2	0.571
	Muscle group x Pulse duration	0.83	4	0.510
**Mean force 50 Hz**	*Muscle group*	5.73	2	**0.004**
	Pulse duration	2.71	2	0.070
	Muscle group x Pulse duration	1.45	4	0.222
**Mean force 100 Hz**	*Muscle group*	8.84	2	**< 0.001**
	Pulse duration	1.68	2	0.190
	Muscle group x Pulse duration	0.60	4	0.663
**Mean force 147 Hz**	*Muscle group*	10.29	2	**< 0.001**
	Pulse duration	0.74	2	0.478
	Muscle group x Pulse duration	0.77	4	0.546
**Discomfort 20 Hz**	*Muscle group*	43.99	2	**< 0.001**
	Pulse duration	0.69	2	0.506
	Muscle group x Pulse duration	0.28	4	0.889
**Discomfort 50 Hz**	*Muscle group*	2.58	2	0.080
	Pulse duration	0.27	2	0.761
	Muscle group x Pulse duration	0.99	4	0.416
**Discomfort 100 Hz**	*Muscle group*	7.30	2	**0.001**
	Pulse duration	0.01	2	0.992
	Muscle group x Pulse duration	0.64	4	0.633
**Discomfort 147 Hz**	*Muscle group*	16.97	2	**< 0.001**
	Pulse duration	0.87	2	0.423
	Muscle group x Pulse duration	0.62	4	0.647

Significant p values are displayed in bold.

Supplementary Table 4. Linear mixed model statistics inter-Muscle group

Fixed effect tests		F	Degrees of freedom	P value
**Extra force 0.2 ms**	*Muscle group*	2.84	2	0.062
	Stim frequency	1.43	3	0.236
	Muscle group x Stim frequency	1.17	6	0.325
**Extra force 1 ms**	*Muscle group*	12.07	2	**< 0.001**
	Stim frequency	1.09	3	0.354
	Muscle group x Stim frequency	1.24	6	0.289
**Extra force 2 ms**	*Muscle group*	6.69	2	**0.002**
	Stim frequency	1.18	3	0.318
	Muscle group x Stim frequency	0.97	6	0.450
**Sustained EMG activity 0.2 ms**	*Muscle group*	13.47	2	**< 0.001**
	Stim frequency	3.87	3	**0.010**
	Muscle group x Stim frequency	1.32	6	0.250
**Sustained EMG activity 1 ms**	*Muscle group*	15.86	2	**< 0.001**
	Stim frequency	5.23	3	**0.002**
	Muscle group x Stim frequency	0.86	6	0.523
**Sustained EMG activity 2 ms**	*Muscle group*	25.50	2	**< 0.001**
	Stim frequency	3.21	3	**0.025**
	Muscle group x Stim frequency	0.95	6	0.461
**Mean force 0.2 ms**	*Muscle group*	2.19	2	0.115
	Stim frequency	1.43	3	0.235
	Muscle group x Stim frequency	0.80	6	0.573
**Mean force 1 ms**	*Muscle group*	11.84	2	**< 0.001**
	Stim frequency	4.08	3	**0.008**
	Muscle group x Stim frequency	1.33	6	0.246

**Mean force 2 ms**	*Muscle group*	9.88	2	< **0.001**
	Stim frequency	3.17	3	**0.026**
	Muscle group x Stim frequency	1.36	6	0.235
**Discomfort 0.2 ms**	*Muscle group*	4.57	2	**0.012**
	Stim frequency	0.42	3	0.739
	Muscle group x Stim frequency	0.99	6	0.433
**Discomfort 1 ms**	*Muscle group*	23.98	2	< **0.001**
	Stim frequency	0.44	3	0.724
	Muscle group x Stim frequency	2.21	6	**0.045**
**Dicomfort 2 ms**	*Muscle group*	17.56	2	< **0.001**
	Stim frequency	0.362	3	0.780
	Muscle group x Stim frequency	2.58	6	**0.020**

Significant p values are displayed in bold.
